# Postnatal depression is associated with detrimental life-long and multi-generational impacts on relationship quality

**DOI:** 10.7717/peerj.4305

**Published:** 2018-02-16

**Authors:** Sarah Myers, Sarah E. Johns

**Affiliations:** School of Anthropology and Conservation, University of Kent, Canterbury, Kent, United Kingdom

**Keywords:** Postnatal depression, Relationships, Bonding, Mother-child, Embodied capital, Grandmothers, Bromley Postnatal Depression Scale, Edinburgh Postnatal Depression Scale, Intergenerational, Life history

## Abstract

Postnatal depression (PND) is known to be associated with a range of detrimental child and adolescent outcomes, resulting from its disruptive impact on mother-child relationship quality. However, until now little has been known about the impact of PND on the longer-term relationships between mothers and their children, and any intergenerational effects this may have. Mother-child relationship quality is of interest from an evolutionary perspective as it plays a role in the accrual of offspring embodied capital, thus affecting offspring quality and offspring’s capacity to subsequently invest in their own children. Relationships with offspring also mediate grandparent-grandchild relations; if PND negatively affects long-term mother–offspring relationship quality, it is also likely to negatively affect grandmaternal investment via reduced grandmother–grandchild relationship quality. Here, we use responses to a retrospective questionnaire study of postmenopausal women, largely from the UK and US, to assess the impact of PND occurring in generation 1 on mother–child relationship quality across the life course of the child (generation 2) with whom it was associated, and also on the relationship quality with grandchildren (generation 3) from that child. Average mother-child relationship quality was lower when the child’s birth was associated with PND. Multi-level regression modelling found that mother-child relationship quality decreased as PND symptom severity increased after controlling for individual effects and a variety of other factors known to influence relationship quality (individual mothers *n* = 296, mother-child dyads *n* = 646). Additionally, intergenerational relationships appear to be affected, with PND negatively associated with grandmother-grandchild relations (individual grandmothers *n* = 125, relations with grandchildren from *n* = 197 grandmother-parent dyads). That PND has long-term detrimental consequences for mother-child relationships, well beyond adolescence, highlights the need for investment in strategies to prevent PND and its cascade of negative multigenerational effects.

## Introduction

Postnatal depression (PND), functionally defined as a major depressive episode occurring within 12 months of giving birth (Stowe, Hostetter & Newport, 2005; Halbreich & Karkun, 2006; Skalkidou et al., 2012), has received a great deal of attention from psychologists due to its association with a range of detrimental outcomes in children (Gelfand & Teti, 1990; Beck, 1998). For instance, maternal PND has been found to correlate with lower infant weight gain and weight faltering during the first four months after birth in the offspring whose birth is associated with the depression (Wright, Parkinson & Drewett, 2006), poorer infant motor development by 15 months (Cornish et al., 2005), stunted growth at two years (Avan et al., 2010), and higher rates of morbidity (when monitored for the first four years) due to gastrointestinal and lower respiratory tract infections (Ban et al., 2010). Additionally, chronic depression in mothers is associated with poorer cardiac function in their children aged 9.5 years (Gump et al., 2009). PND is also implicated in poorer child behavioural and cognitive outcomes, correlating with, for example, behavioural problems at 18 months (Murray, 1992), reduced interpersonal functioning at 19 months (Stein et al., 1991), behavioural problems at 2 years (Avan et al., 2010), and lower cognitive ability at age four (Cogill et al., 1986). A review of the quantitative and qualitative literature also points to consistent findings of correlations with poorer language and intelligence development, as measured by intelligence quotient (IQ), throughout childhood (O’Hara & McCabe, 2013). PND is thought to have a disruptive impact on mother-infant bonding and attachment (Beck, 1995; Murray et al., 1996; Coyl, Roggman & Newland, 2002; Moehler et al., 2006); postnatally depressed mothers show heightened self-focus (Salmela-Aro et al., 2001), and are more likely to exhibit a range of potentially problematic parenting behaviours (for a review see O’Hara & McCabe, 2013). The negative effect on parenting is suggested to mediate the relationship between PND and subsequent child development, potentially alongside shared environmental or genetic factors influencing both PND aetiology and child development outcomes (Murray & Cooper, 1997).

The number of long-term studies on the impact of PND are small but growing (Sanger et al., 2015) and research now extends to the adolescent period; for example, PND experienced by the mother is correlated with an increased incidence of teenage depressive and anxiety disorders (Halligan et al., 2007; Murray et al., 2011), higher cortisol levels (Halligan et al., 2004), lower IQ (Hay et al., 2008), and lower academic attainment at age 16 in the child (Murray et al., 2010; Pearson et al., 2016). PND has also been associated with increased rates of marital discord and depression in partners (Boath, Pryce & Cox, 1998; Burke, 2003), which may further affect developmental outcomes in children from families where these issues arise.

However, little is known about the impact of PND on the longer-term relationships between mothers and their children. Lower mother–offspring relationship quality is linked to reduced offspring self-esteem and social competence (Kim & Cicchetti, 2004), which has potential consequences such as being less able to achieve high social status (Rudolph, Hammen & Burge, 1995) or succeed in the job market (Baron & Markman, 2003). Such effects are of theoretical interest from an evolutionary perspective as they indicate that mother–offspring relationship quality plays a role in the accrual of offspring embodied capital and hence offspring quality. Embodied capital refers to the attributes and resources an individual has available for conversion into biological fitness enhancing commodities (Kaplan et al., 1995), i.e., the attributes and resources that influence whether an individual succeeds in attracting a mate, and the resources available for investing in resulting children. Parents are thought to make investments in their children’s embodied capital based on their own capital and the perceived likelihood that their investments will pay off in the form of grandchildren. Therefore, mother–child relationships are of interest in both terms of their effect on offspring quality and offspring’s capacity to subsequently invest in their own children.

The influence of PND on intergenerational relationships is also of evolutionary interest, yet is currently unknown. Grandmothers are thought to play an important role in human evolution; evolutionary anthropologists have repeatedly highlighted the importance of grandmothers in enhancing child survival (Sear & Mace, 2008) and their influence on the reproductive trade-offs of their offspring (Hawkes & Coxworth, 2013) in pre-industrial contexts. A small body of literature also indicates that grandparents can enhance grandchild development and growth via socio-emotional support in low-risk industrialised settings (Coall & Hertwig, 2010). In Western contexts, it has been demonstrated that relationships with offspring mediate grandparent-grandchild relations (Michalski & Shackelford, 2005), positively correlating with grandparental involvement (a form on investment) with their grandchildren (Barnett et al., 2010). If PND negatively affects mother–offspring relationship quality, it is also likely to negatively affect grandmaternal investment from the grandmother who experienced PND via reduced grandmother–grandchild relationship quality. Grandparents make investments that influence their children’s reproductive and parental investment decisions even in modern, developed settings (Coall & Hertwig, 2010; Coall & Hertwig, 2011). For instance, in the UK contact frequency with grandparents predicts parents’ continuation of childbearing (Tanskanen et al., 2014) and the educational achievement of their children in the first year of school (Tanskanen & Danielsbacka, 2016; Tanskanen, 2017), contact frequency with grandmothers in the UK is associated with lower levels of breastfeeding in mothers (Emmott & Mace, 2015), and, in Bulgaria, supportive residential grandparents were found to protect adolescents from external stressors which otherwise correlated with increased depressive symptoms (Botcheva & Feldman, 2004).

Here we present results from a retrospective survey study, designed to gather the complete reproductive histories of postmenopausal women (generation 1), along with measures of their history of PND, depressive tendencies more generally, demographic characteristics, the reproductive histories of their offspring (generation 2) so far, and the relationship quality they have with their children (generation 2) and grandchildren (generation 3). The ages of the children of respondents (generation 2) ranged from 8 to 48 years, with an average age of 29 years, allowing assessment of the impact of PND over a longer period, to the best of our knowledge, than previous studies. We first test the hypothesis that (1) *Mothers (generation 1) who experienced PND will have lower quality relationships throughout their lives with the children (generation 2) with whom the depression was experienced* and then, by extension, (2) *grandmother–grandchild relationship quality will be lower when the grandchildren (generation 3) come from a child (generation 2) whose birth was associated with PND*.

## Materials and Methods

### Data collection

A retrospective questionnaire was used to collect the complete reproductive histories of post-menopausal women. Respondents reported details about every birth they had experienced, separately and in chronological order, and were assessed on a number of demographic and psychological measures, including three different measures of PND and a measure of mother–child relationship quality. Participants were recruited via advertising in newsletters and social media channels of UK-wide branches of the Women’s Institute (a voluntary organisation providing social and educational opportunities for its 212,000 members, 93% of whom are aged 45 and over (National Federation of Women’s Institutes, 2014), social media aimed at older women, and the alumni networks of two UK universities. The survey was conducted online using the SurveyGizmo platform and, due to the sensitive nature of the information requested, participants remained anonymous with the exception of their IP address, which was collected to control for multiple responses from the same address, to minimise inaccurate reporting. Valid responses from 305 participants were received; the sample is the result of convenience sampling and was the maximum size obtainable within the time limits of the study (participant recruitment took place between late November 2013 and early May 2014). Data with which to replicate the analysis are available in the Supplemental Information 1. The study was approved by the Research and Ethics committee of the School of Anthropology and Conservation at the University of Kent, UK. All participants read a statement regarding the aims and content of the questionnaire, namely to “gain a greater understanding of how women experience early motherhood and the long-term effects these experiences have on women” (for full details of the information presented to participants see the Supplemental Information 1). However, the underlying hypotheses guiding the survey design were not revealed to the participants. The potentially sensitive nature of the questions surrounding postnatal emotional experience was highlighted, and by proceeding participants were deemed to have given written informed consent. Participants were informed that they could withdraw from the study at any time, in which case that their responses would be destroyed. No incentives were given in exchange for participation.

### Postnatal depression

Women were asked to self-report their diagnostic history of PND, giving a categorical measure of *PND incidence* based on actual diagnosis by a medical professional after each birth (PND No/PND Yes). It was anticipated that this sample size would be small on the grounds that PND is chronically under-diagnosed (Paulden, Palmer & Hewitt, 2009), so two retrospective screening measures for PND, the Bromley Postnatal Depression Scale (BPDS) (Stein & Van den Akker, 1992) and a modified version of the Edinburgh Postnatal Depression Scale (EPDS) (Cox, Holden & Sagovsky, 1987), were also completed for each reported birth.

The BPDS requires participants to read a statement regarding depressive symptoms and answer a question regarding whether they experienced such symptoms; if the answer is affirmative the symptom duration is recorded, with symptoms persisting for over a month being indicative that PND occurred. The BPDS was used to determine a categorical measure of *PND incidence* at a given birth (PND No/PND Yes). The BPDS is specifically designed to retrospectively assess PND symptoms (Stein & Van den Akker, 1992) and has been used in previously published studies assessing recall over similar durations (McLaren et al., 2007; Séjourné et al., 2011), yet it provides no scope for assessing symptom severity. For this reason a modified version of the EPDS is used.

The 30 point EPDS is the most commonly used PND screen (Boyd, Le & Somberg, 2005). Questions were modified to the past tense and participants were requested to reflect back on the first year after each of their births. An EPDS score for each birth was calculated and was used as a continuous measure of *PND severity* at a given birth. The EPDS was also used to determine a categorical measure of *PND incidence* after each birth by using a cut-off score of 12 (PND No/PND Yes), where 12 or above indicated PND had occurred (this methodology is very similar to that employed by Meltzer-Brody et al., 2013).

The *PND severity* score was employed in the regression analysis to avoid issues potentially stemming from the choice of cut-off in either the EPDS or BPDS. For a more expansive discussion and justification of this methodology see Myers, Burger & Johns (2016). In analyses requiring a categorical measure of *PND incidence* we employ a combined measure in which PND is deemed to have occurred if an actual diagnosis was received, or a birth met the criteria for PND on *both* the EPDS and BPDS categorical screening measures.

### Relationship quality

#### Mother–child relationship quality

Mother–child relationship quality was determined by the Positive Affect Index (PAI) (Bengtson & Schrader, 1982) which measures subjective relationship solidarity. The index is made of two parts assessing a mother’s feelings *towards* her child and her perception of her child’s feelings *about* her. Subjective solidarity is measured along five dimensions of affect: (1) understanding, (2) fairness, (3) trust, (4) respect, and (5) affection. Degree of solidarity was rated on a Likert scale of 1 (not well) to 5 (extremely well) following Birditt, Rott & Fingerman (2009); the index then provides three continuous measures: a score out of 25 for feelings *towards*, a score out of 25 for perceptions *about*, and a combined measure of overall *mother–child relationship quality* scored out of 50 which was used in the analysis. Where multiple children were born at the same birth event, mothers reported average relationship quality between her and the children from the birth (*N* = 10 twins).

#### Grandmother–grandchild relationship quality

Those participants who reported that they had grandchildren from one or more of their children were asked to rate how emotionally close they felt with each grandchild, on Likert scales of 1 (*very close*) to 5 (*not at all close*), and also whether they felt the relationship they have with their child positively or negatively impacts their relationship with their grandchild/grandchildren, on Likert scales of 1 (*very positive*) to 5 (*very negative*). These scales were subsequently condensed into three categories due to sample size constraints.

### Other variables

While PND was our primary variable of interest, a range of other factors are known to influence mother–child relationships. As such the following measures were taken to avoid confounding effects:

#### General depression

*Current depression* at the time of survey completion may adversely affect the recall of past events, as well as influence relationship quality, so the Beck Depression Index-Short Form (BDI-SF) (Beck & Steer, 1993) controlled for respondent’s current emotional state. A cut-off of 5 and above was used to indicate depression following the cut-offs determined by Stukenberg, Dura & Kiecolt-Glaser’s (1990) validation of the scale in an elderly non-clinical community.

To control for any negative affective tendencies throughout the respondent’s life course we used the short version of the Depression Anxiety Stress Scales (DASS)(Lovibond & Lovibond, 1995). The “trait wording” version of the DASS was used, which asks respondents to report how they feel in general, as opposed to the “state wording” version which refers to feelings in the past week (Lovibond, 1998). The DASS is designed to be compared to normative population data (Crawford & Henry, 2003; Psychology Foundation of Australia, 2013) and is split into separate sections for each emotional state, each giving a score out of 63. The aggregated score was used as a measure of *general tendency towards depression, anxiety, and stress (DAS)*.

#### Birth characteristics

Birth trauma is an increasingly recognised issue (Beck et al., 2011) and, like PND, may have long-lasting impacts. Respondents were asked for each birth whether their *emotional experience of birth* was ‘positive’, ‘negative’, or ‘mixed’.

Respondents also reported whether during birth they experienced no complications, minor complications, and major complications, to determine a measure of the *occurrence of birth complications*.

Low infant birth weight and infant health issues have been found to decrease maternal investment in humans (Bereczkei, Hofer & Ivan, 2000; Bereczkei, 2001). Participants were asked whether their child’s birth weight was ‘low’, ‘normal’, or ‘high’; *infant birth weight* was subsequently collapsed into categories of ‘normal’ and ‘not normal’ for the analysis, as the sample size of low and high were small and high birth weight infants are also at increased risk of future morbidity (Ørskou et al., 2003; Danielzik et al., 2004; Harder et al., 2007). A categorical measure of *infant health issues* was determined by asking participants whether their child had any serious health issues in their first year post-birth.

#### Breastfeeding

Breastfeeding has been linked to enhanced infant bonding and attachment (Else-Quest, Hyde & Clark, 2003; Britton, Britton & Gronwaldt, 2006); respondents were asked to report whether or not they breastfed each of their individual children, creating a categorical measure of *breastfeeding occurrence* (Yes/No).

#### Social environment

Social support has been shown to have a positive impact on mother–offspring interaction and buffer the negative impact of stress (Crnic et al., 1983), as such a respondent’s perceived *level of support* during the first year after each birth from (1) the offspring’s *father*, (2) her *family*, and (3) her *friends*, (4) whether her own mother (generation 0) was alive at the time of her first giving birth, and if so, (5) the perceived level of support available to her specifically from her *mother*. Level of support was rated on a scale of 1 (*low*) to 3 (*high*) or for maternal support 0 (*none*—when the mother wasn’t alive at first birth) to 3 (*high*).

Stress and depression are associated with the perception of social stigma (Slavich et al., 2010a; Slavich et al., 2010b), thus stigma surrounding mothering is likely to increase negative affect and may impact mother–child relations. Respondents were also asked if they experienced *social pressure* to be a ‘good mother’, with ‘yes’ or ‘no’ response options.

#### Demographics

Respondents reported their dates of birth and those of their offspring, from which the mother’s *age at birth* and *child’s current age* at the time of survey completion were calculated. The latter allows us to control for the possibility that mother–child relations may improve with time since any postnatal depressive event.

*Socioeconomic status (SES)* during respondents’ childbearing years was determined by the Social Class Based on Occupation method (CeLSIUS, 2007) and categorised as either high (professional), medium (managerial and technical), or low (skilled non-manual, skilled manual, partly-skilled, and unskilled).

### Sample characteristics

**Figure 1:**
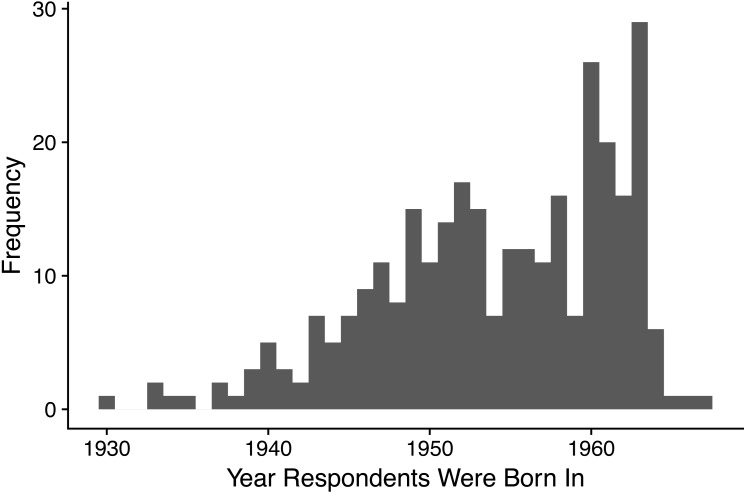
Histogram showing the frequency distribution of years in which respondents were born. All respondents were born between 1930 and 1967, *n* = 305.

Respondents (generation 1) had a mean age of 59.9 years (SD 7.4) and were born between 1930 and 1967 (Fig. 1). The majority of respondents did their childrearing in the UK (74.5%), followed by North America (12.9%), and were of high to medium SES (‘professional’ 67.9%, ‘managerial and technical’ 20.7%). Respondents gave birth to a mean of 2.2 children (SD 0.8, range 1–6). An actual diagnosis of PND, at at least one birth, was received by 40 women (13.1%), while 72 women (23.6%) met the criteria for PND according to the BPDS at least once, and 110 women (36.1%) met the cut-off for PND according to the EPDS at least once. Data on *mother–child relationship quality* was reported for 666 offspring (generation 2) (for 15 offspring there was non-systematic missing data); the mean PAI score was 42.8 (SD 5.6, range 17–50) and the mean age of these offspring was 29.1 years (SD 9.7, range 8–48 years). 125 respondents (generation 1) had 373 grandchildren (generation 3) born to 197 children (generation 2).

### Statistical analysis

All mother–child dyads and relations with grandchildren from grandmother-parent dyads, for whom data on the relevant variables was available, were included within the analyses.

(1) Mothers (generation 1) who experienced PND will have lower quality relationships throughout their lives with the children (generation 2) with whom the depression was experienced

*1.i* First, to assess whether experiencing PND predicts later mother–child relationship quality Wilcoxon signed-rank tests were performed on relationship quality (PAI score) split by PND incidence after child’s birth (*n* = 666) (for the separate results of each of the three different PND measures see the R code in the Supplementary Information). The effect size for this effect is reported as *r*. A non-parametric test was used due to the non-normal distribution of the PAI scores.

*1.ii* Next, we assessed whether PND severity (EPDS score) predicts mother–child relationship quality (individual mothers *n* = 296, mother–child dyads *n* = 646) once other factors that may influence relationship quality are considered. A multilevel model was used to account for correlations within the data reported by individuals (i.e., to account for the fact that mothers may have multiple children), with mother–child relationship quality (PAI score) acting as the outcome variable. Exploratory analysis of a model with all available variables found many to have coefficients whose 95% confidence intervals overlapped zero (see R code in the Supplemental Information 1), so instead we present a model based on the following modelling strategy: First, we ascertained that a random intercept, allowing intercepts to vary by mother, improved upon an intercept only model using a chi-square test for reduction in the −2 log likelihood (−2LL) with *α* <0.05. To this random intercept model we the added each of our available variables separately (dummy variables were used to convert categorical variables with 3 or more categories into binary variables) and ranked the resulting models according to their Akaike Information Criterion (AIC) value. Next we constructed a base model, adding the variables current depression (which may influence reporting of relationship quality) and child’s current age (as relationship quality may vary across age cohorts) to our random intercept model, checking that this reduced the -2LL. To this base model we then added our available variables one by one, from lowest AIC to highest, retaining them if they significantly reduced the -2LL relative to the previous iteration of the model, until all variables had been tested, and finally add random slopes. All models were estimated using the maximum likelihood method; the results for each model can be found as part of the R code in the Supplemental Information 1. We present the results of the final model, and a PND only model for comparison, along with Nakagawa and Schielzeth’s pseudo R^2^s for the fixed and conditional effects (Johnson, 2014; Nakagawa & Schielzeth, 2013). The 95% confidence intervals of the coefficients are bootstrapped to counter potential heteroscedasticity indicated by the plotting of residuals from a single level version of the model (Field, 2013).

(*2*) *grandmother–grandchild relationship quality will be lower when the grandchildren (generation 3) come from a child (generation 2) whose birth was associated with PND*

*2.i* Fisher’s exact tests were performed on the distributions of the nature of the impact the mother–child relationship has on grandmother–grandchild relations, split by whether the birth of the grandchild’s parent was associated with PND (individual grandmothers *n* = 125, relations with grandchildren from *n* = 197 grandmother-parent dyads). The effect size of this association is measured using a bias corrected Cramér’s *V*.

*2.ii* Fisher’s exact tests were performed on the distributions of reported relationship quality with grandchildren, split by whether the birth of the grandchildren’s parent was associated with PND (individual grandmothers *n* = 125, relations with grandchildren from *n* = 197 grandmother–parent dyads). The effect size of this association is measured using a bias corrected Cramér’s *V*.

All statistical analysis was conducted using R version 3.2.3 (for details of the specific packages and all code used see Supplemental Information 1).

## Results

(*1) Mothers (generation 1) who experienced PND will have lower quality relationships throughout their lives with the children (generation 2) with whom the depression was experienced*

**Figure 2:**
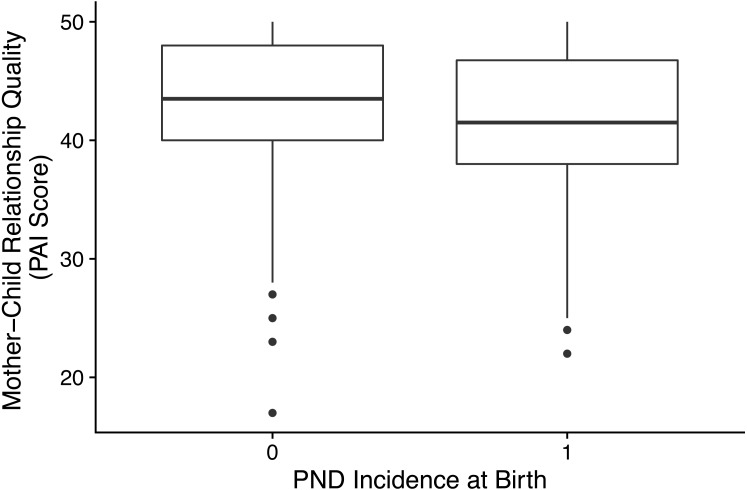
Box plot showing the distribution of mother-child relationship quality (PAI score) split by PND incidence. The centre line indicates the median, the box limits indicate the 25th and 75th percentiles, the whiskers are 1.5× the interquartile range or the maximum value, the points indicate outliers. 0 = No PND (*n* = 578), median = 44, outliers = 5; 1 = PND (*n* = 88), median = 41, outliers = 2.

*1.i* mother–child relationship quality (PAI score) was statistically significantly lower when the child’s birth (generation 2) was associated with PND (median = 43.5, *n* = 564) compared to when the child’s birth was PND free (median = 41.5, *n* = 102): *W* = 33, 656, *p* = 0.006, *r* =  − 0.106. The distribution of PAI scores by PND incidence can be seen in Fig. 2).

**Figure 3:**
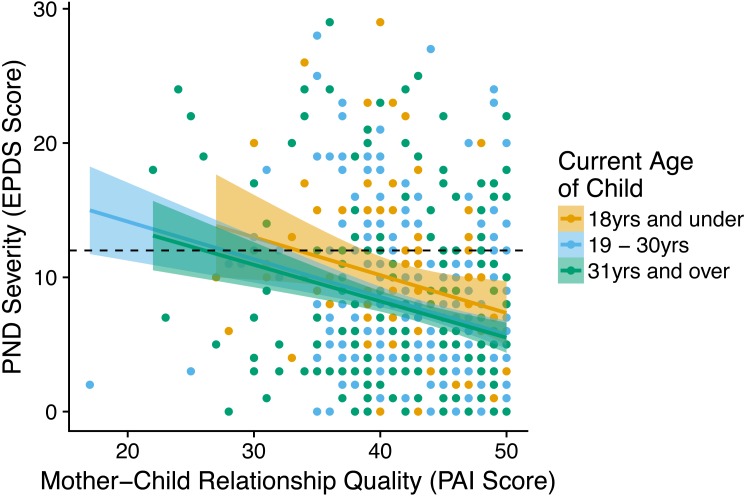
Graph showing the relationship between the PND severity (EPDS score) at a given birth and the mother-child relationship quality (PAI score) with the child from that birth. Children split into age categories: children and adolescents (18 years of age and under *n* = 91), early adulthood (19–30 years *n* = 289), and later adulthood (31 years and over *n* = 283). The dashed line on the y-axis indicates the cut-off for PND, with PND classed as having occurred in cases above the line. Shaded areas reflect 95% confidence intervals.

**Table 1:** Results of multilevel regression showing the effect of PND severity (EPDS score) on mother-child relationship quality (PAI score). Legend: Individual mothers *N* = 296, mother-child dyads *N* = 646. Confidence intervals (CI) not overlapping zero indicated in bold; CIs reflect the result of percentile bootstrapping based on 1,000 samples. Pseudo R2s calculated using Nakagawa & Schielzeth’s method. AIC, Akaike information criterion.

Variable		Coefficient	SE	Lower 95% CI	Upper 95% CI	Pseudo *R*^2^		AIC
						Fixed effects	Conditional effects	
***PND only model—individual mothersn*** = **296**, ***mother-child dyadsn*** = **646**								
PND severity		−0.175	0.037	−0.249	−0.104	0.038	0.456	3932.727
(Intercept)		44.252	0.402	43.446	45.044			
***Final model—individual mothersn*** = **296**, ***mother-child dyadsn*** = **646**								
PND severity		−0.117	0.045	−0.202	−0.024	0.117	0.513	3904.970
Current depression	No (ref)	–	–	–	–			
	Yes	−0.250	0.637	−1.539	0.898			
Current age of child		0.002	0.025	−0.047	0.050			
Support from own mother	High (ref)	–	–	–	–			
	Medium	−1.722	0.729	−3.242	−0.321			
Support from own mother	High (ref)	–	–	–	–			
	Low	−1.689	0.611	−2.853	−0.451			
Support from own mother	High (ref)	–	–	–	–			
	None	1.191	0.888	−0.634	2.988			
(Intercept)		45.855	0.957	44.046	47.758			

*1.ii* As PND severity (EPDS score) increased mother–child relationship quality (PAI score) decreased across age ranges (Fig. 3). Multilevel analysis showed this relationship remained after controlling for both individual effects of the mothers (generation 1) alone and with other factors (Table 1); a woman’s (generation 1) PND symptom severity at a given birth was negatively correlated with her relationship quality with the child from that birth (generation 2). The PND only model found that controlling for only PND severity accounted for 4% of the variance in mother–child relationship quality.

The following factors, which did not vary between a mother’s (generation 1) births, also negatively correlated with mother–child relationship quality (Table 1): having a general tendency towards DAS, and having medium to low levels of support from one’s own mother compared to having high levels of support from her. The age of the child (generation 2) had no impact on the relationship quality between mother (generation 1) and child (generation 2), nor did it interact with PND symptom severity (see R code in the Supplemental Information 1).

(*2*) *Grandmother–grandchild relationship quality will be lower when the grandchildren (generation 3) come from a child (generation 2) whose birth was associated with PND*

*2.1* The relationship a mother (generation 1) has with her child (generation 2) was less likely to have a positive impact on her relationship with her grandchildren (generation 3) from that child if the child’s birth (generation 2) was associated with PND (Table 2): Fisher’s exact test = 5.227, *p* = 0.064 (two-tailed), Cramér’s *V* = 0.133.

*2.ii* Relationships with grandchildren (generation 3) were of lower emotional closeness when the birth of the grandchild’s parent (generation 2) was associated with PND (Table 2): Fisher’s exact test = 9.845, *p* = 0.006 (two-tailed), Cramér’s *V* = 0.222.

## Discussion

The detrimental consequences of PND on mother–child relationships over the short-term are well documented, but until now little was known about any of the longer term impacts. This is the first piece of research to document that PND is associated with a reduction of mother–child relationship quality into the offspring’s adult years. Maternal (generation 1) self-reported mother–child relationship quality decreased as PND symptom severity increased, after controlling for a variety of other factors likely to influence relationship quality. The level of support from maternal grandmothers (generation 0), when alive during their daughter’s (generation 1) childbearing years, also positively correlated with mother–child relationship quality. Additionally intergenerational relationships also appear to be affected, with grandmaternal (generation 1) self-reported grandmother–grandchild relations more likely to be negatively categorised if PND occurred at the birth of the parent (generation 2), indicating a hitherto unknown consequence of PND.

**Table 2:** The distributions of grandmother-grandchild emotional closeness ratings and the impact mother-child relations have on grandmother-grandchild relations split by PND incidence. PND incidence determined by either an actual diagnosis or meeting the criteria for PND on both the EPDS and BPDS (PND No *N* = 161, PND Yes *N* = 36). Individual grandmothers *n* = 125, relations with grandchildren from *n* = 197 grandmother-parent dyads.

PND incidence		Impact of mother-child relations on grandmother-grandchild relations (observed/expected)			Emotional closeness with grandchildren (observed/expected)		
		Very positive-positive	No impact	Negative- very negative	Very close- close	Moderately close	Quite close- not at all
**PND**	No	137.0/133.2	21.0/22.9	3.0/4.9	119.0/114.4	27.0/25.3	15.0/21.2
	Yes	26.0/29.8	7.0/5.1	3.0/1.1	21.0/25.6	4.0/5.7	11/4.8

Until recently, widespread screening for PND, in the UK at least, was considered to not be cost effective (Paulden, Palmer & Hewitt, 2009); however, there have been recent political moves in the UK to improve spending on maternal mental services (Campbell, 2016), and in 2016 the US Preventative Services Taskforce recommended population wide screening for depression in pregnant and postpartum women (O’Connor et al., 2016). Results pointing to the long-term impact of PND add impetus to the necessity of investing in preventative strategies, rather than simply identifying women once they are already depressed. A wide range of risk factors for PND are well documented (for a review see Yim et al., 2015) and successful intervention strategies for high-risk groups do exist (Austin et al., 2008; Dennis et al., 2009).

Most crucially, our results also suggest that while maternal tendency towards depression, anxiety, and stress, more generally across the life course reduces mother–child relationship quality, the negative effect of PND is specific to the child whose birth the PND was associated with, rather than being related to a lowering of relationship quality with all of a mother’s children. A proximate level explanation for this may be that the memory of PND has a permanent detrimental impact on relations with the child with whom it was associated. However, an evolutionary perspective suggests a potential ultimate level explanation: if mother–child relationship quality can be taken as a proxy for maternal investment, or indeed as a form of investment, this suggests that factors in early infancy may affect the pay-offs of maternal investment in terms of offspring quality which set the course of life-long investment levels.

Mother–child and grandmother–grandchild relationship quality are also likely to influence maternal/grandmaternal subsidy of offspring parenting costs, in turn impacting the investment received by grandchildren. Respondents (generation 1) were more likely, at a level approaching significance, to rate their relationship with their child (generation 2) as having a negative effect on their relationship with their grandchildren (generation 3) from that child if they had PND in association with the child’s birth. They were also more likely to report feeling lower emotional closeness with grandchildren (generation 3) when PND occurred in association with the birth of the parent (generation 2) of the grandchildren. This effect is likely to be mediated by the reduced mother–child relationship quality experienced in association with PND (Michalski & Shackelford, 2005); in the United States at least, lower quality grandmother-parent relationships predict lower levels of grandmother investment both in terms of contact frequency with grandchildren and help raising them (Barnett et al., 2010).

There is a large sociological and public health literature highlighting the importance of maternal grandmothers in helping their daughters raise their offspring (for a review see Scelza, 2011). Evolutionary anthropologists have also highlighted, largely in natural fertility populations, the importance of the support of grandmothers for maternal investment and subsequent child outcomes, with the presence of maternal grandmothers generally improving child survival rates (Sear & Mace, 2008). Thus, our finding that mothers (generation 1) who report higher levels of support from their own mothers (generation 0) tend to report higher quality relationships with their children (generation 2) is unsurprising.

It must be noted that the size of the effects documented here are small; however, given more immediate events and conditions are to be expected to have a large effect on the reporting of relationship quality, this is unsurprising. Nonetheless, these results indicate that conditions early in a mother and child’s relationship may have long-lasting consequences.

### Limitations

The primary limitation of this study is that the measure of mother–child relationship quality relied on maternal self-reported feelings; while these varied between a mother’s children, we have no data on whether the different children of a mother perceived any variation in the relationships between themselves and their mother’s compared to their siblings. Nor do we have data on how differences in mother–child or grandmother–grandchild relationship quality manifested in terms of interactions with, or investment of, more tangible resources in (grand)children, and this is an avenue for future research. Our sample, being drawn from industrialised populations, is obviously unrepresentative of the human species as a whole (Henrich, Heine & Norenzayan, 2010), thus the cross-cultural and historical applicability of these findings is uncertain. This being the case, we refrain from speculating as to whether or not these findings have implications for debates surrounding the evolutionary origins of PND (Myers, Burger & Johns, 2016; Hagen & Thornhill, 2017; Myers, Burger & Johns, 2017). We have no data with which to test for ethnic differences in these relationships; ethnicity is an ambiguous means by which to categorise individuals (Bhopal, 2004). Any ethnic differences found would difficult to interpret and likely be the result of correlations between ethnicity and other factors, such as SES or access to kin networks. Finally, PND was largely assessed retrospectively; we know of no data currently enabling prospective analysis of the impact of PND over a similar duration of time. However, longitudinal cohort studies of individuals from infancy which also recorded maternal mental health, such as the UK’s Millennium Cohort Study conducted by the Centre for Longitudinal Studies, may allow for replication of these results in the near future.

## Conclusions

PND appears to have long-term detrimental consequences for mother–child relationships well beyond adolescence. Inter-generational effects of PND were also indicated, with PND not only associated with reduced emotional closeness with children but with the grandchildren of these children as well. As such, these results indicate that PND is implicated in a cascade of negative multigenerational effects and suggest that investment in PND preventative measures may not only improve relationships between mothers and their children in the short-term, but will also improve family relationships, closeness, and support well into the future.

##  Supplemental Information

10.7717/peerj.4305/supp-1 Supplemental Information 1 Data, variables, and R code for the analyses of the impact of post-natal depression on relationship quality (A) Complete dataset, (B) all variables used in the analyses, including detailed variable descriptors and value codes, (C) R code for the Wilcoxon’s rank-sum test, (D) R code for the multilevel analysis, (E) R code for the Fisher’s Exact test.

## Additional Information and Declarations

### Data Availability

The following information was supplied regarding data availability: The raw data has been provided as Supplemental Information 1.
